# Yessotoxin as a Tool to Study Induction of Multiple Cell Death Pathways 

**DOI:** 10.3390/toxins4070568

**Published:** 2012-07-23

**Authors:** Mónica Suárez Korsnes

**Affiliations:** The Norwegian School of Veterinary Science, P.O. Box 8146 Dep., N-0033 Oslo, Norway; Email: monica.suarez.korsnes@nvh.no; Tel.: +47-229-645-00

**Keywords:** apoptosis, paraptosis, multiple signalling pathways, yessotoxin, cancer, neurodegenerative diseases

## Abstract

This work proposes to use the marine algal toxin yessotoxin (YTX) to establish reference model experiments to explore medically valuable effects from induction of multiple cell death pathways. YTX is one of few toxins reported to make such induction. It is a small molecule compound which at low concentrations can induce apoptosis in primary cultures, many types of cells and cell lines. It can also induce a non-apoptotic form of programmed cell death in BC3H1 myoblast cell lines. The present contribution reviews arguments that this type of induction may have principal interest outside this particular example. One principal effect of medical interest may be that cancer cells will not so easily adapt to the synergistic effects from induction of more than one death pathway as compared to induction of only apoptosis.

## 1. Introduction

Programmed cell death mechanisms are significant for development, maintenance and self-regulation of multicellular organisms [1]. Cell-intrinsic regulation exists in the diverse cell death modalities that occur under different physiological and pathological conditions. This regulation depends on death-inducing stimulus. Several mediators, organelles and cellular processes can take part in and activate a diverse cascade of signalling pathways that may interact or cross-talk [2]. Classification of programmed cell death mechanisms are developing and new cell death modalities often appear without a clear reference to precise biochemical mechanisms [3,4]. 

Galluzzi *et al.* [4] recently proposed molecular definitions of a comprehensive and detailed set of cell death subroutines based on measurable biochemical features. They argue that their classification must be interpreted with caution, especially when single parameters are under investigation. The current knowledge about programmed cell death seems often to be associated with morphological criteria and in terms of simpler terminology as compared to Galluzzi *et al.* [4]. Morphologically relatively uniform sets of observations have been important for recognition of different forms of cell death [5]. These traits can be evaluated using light, fluorescence and electron microscopy. Biochemical markers such as caspase activation, cleavage of caspase substrates and related target proteins, DNA fragmentation and changes in the membrane asymmetry can be measured by immunohistochemistry and immunocytochemistry methods. The choice of the methodology to identify cell death mechanisms will depend on the experimental model. Combination of several methods is recommended for proper evaluation of different cell death modalities. 

Programmed cell death is an integral part of complex living systems which typically possess redundancy and a capacity of adaptation. Different biochemical mechanisms within programmed cell death may overlap or act as hidden backups for events to take place. Such systems may not in practice be fully observable. Reference model experiments and preliminary generic views may though support their description. 

The present work provides photographic illustrations indicating diversity in how cells can respond to insults affecting cell death programmes. Such variations may be important for understanding cell death mechanisms. It reflects redundancy and plasticity of cell signalling pathways. 

Programmed cell death mechanisms vary and they are context dependent [6]. Apoptosis is an active gene regulated programmed cell death mechanism executed by caspases [7,8,9,10]. It is characterised by activation of a sequence of cellular, morphological and biochemical changes. These changes include cell shrinkage, compression of cytoplasmatic organelles, dilatation of the endoplasmic reticulum, chromatin condensation, membrane blebbing and DNA fragmentation recognised as a “DNA ladder” [11]. Apoptotic inducers can, however, initiate programmed cell death mechanisms which are independent on caspase activation [6]. These other types of programmed cell death mechanisms such as paraptosis, autophagy and mitotic catastrophe can operate in absence and independent of caspase activation [1]. Caspase-independent cell death programs are activated when caspase-mediated pathways are inhibited or fail to function [1,6]. Furthermore, caspase-independent cell death mechanisms utilise signalling mediators that are also involved in apoptosis induction [6]. 

Knowledge about the distinct regulatory signalling pathways that can be induced during distinct cell death modalities helps to understand the development of some diseases. Dysregulation or defects in execution of programmed cell death mechanisms can cause cancer and neurodegenerative diseases. Disruption of apoptotic functions can in some cases accelerate transformation of a normal cell into a tumour cell [12]. Tumours that succeed in progressing to states of high-grade malignancy and resistance to therapy may do so by overcoming the apoptotic pathway [13,14]. 

Yessotoxin (YTX) is a marine algal toxin that can induce programmed cell death at nanomolar concentrations in different model systems [15,16,17,18,19,20,21,22]. It affects cellular systems in a cell-specific manner. The knowledge about induction of programmed cell death mechanisms and their cross-talk under YTX exposure is substantially incomplete [22]. YTX can also induce a non-apoptotic form of programmed cell death termed paraptosis-like cell death [23]. This type of cell death fails to fulfill the criteria for apoptosis. It is insensitive to a broad range of caspase inhibitors and it operates in a caspase-independent form, although caspase-9 can be activated in an Apaf-1 independent manner [24]. YTX may therefore be of interest in the search for vulnerabilities of cancer cells which are resistant to apoptotic inducers. 

Administration of two different insults inducing for example apoptosis and paraptosis, respectively, may not have the same capacity to kill cancer cells as compared to an insult that can induce both apoptosis and paraptosis. It is presumably harder for a cell to survive after exposure to multiple simultaneous death-inducing stimulus than it is to withstand similar but non-simultaneous ones (as would be the case if the cells where subject to two different drugs with different response/exposure times). A design of a cancer drug may utilise this idea. Therefore, elucidation of the molecular mechanisms and biochemical markers implicated in multiple forms of programmed cell death induced by yessotoxin may help to develop therapeutic approaches related to conditions characterised by excessive cell death or by excessive cell death accumulation. 

## 2. Programmed Cell Death in Disease Models

Apoptotic cell death pathways and their biochemical mediators and regulators are relatively known. However, similar detailed knowledge is missing for non-apoptotic forms of programmed cell death mechanisms [25,26,27]. These programmes operate in a caspase-independent manner and they are not affected by apoptosis inhibitors [24,28]. Non-apoptotic forms of cell death are associated with a variety of neurodegenerative and neoplastic conditions [24]. However, accumulated evidence suggests that under neurodegenerative diseases, all types of programmed cell death phenotypes can take place [29]. 

Activation of both caspase-dependent and caspase-independent signalling pathways under the same death stimuli has been reported in cases where deletion of damaged cells needs execution of complementary programmes to assure cell death [30,31]. Apoptotic and non-apoptotic cell death programmes seem in general often to coexist [32]. Activation of multiple cell death programmes may limit action of cancer cells due to cross-fire effects, or it may act as a default mechanism in neurodegenerative conditions when caspases are inhibited or when caspase-dependent and -independent pathways are activated in parallel [33,34,35]. 

Non-apoptotic caspase-independent cell death programmes may activate in an apoptotic resistant cancer cell. Such programmes may employ death-inducing proteases such as calpains and cathepsins. These proteases can execute cell death independently of the apoptotic machinery, and they may take a dominant role in the progression of tumour development in some cell types by triggering the TNF-cell death signalling pathway [36,37]. Non-apoptotic forms of cell death may also affect tissue reorganisation, regeneration in the nervous system and induce immunological reactions in tumours [35,38]. 

Alternative cell death signalling pathways can ensure safe elimination of unwanted cells. This redundancy of cell removal may protect organisms against development of diseases such as cancer where huge numbers of mutations and failed cell divisions occur during the life span [1]. Cancer cells may inactivate key components for apoptosis such as the p53 pathway, the extrinsic cascade, induced loss of pro-apoptotic proteins, or induced activation of the P13K-Akt/PKB pathway. Cancer cells which develop into aggressive tumours may not have lost the ability to induce apoptosis. However, they may not trigger sufficient death signalling cascades to stop tumour development [35]. They can combine cellular factors to avoid the action of anti-cancer drugs, although drug resistance mechanisms are quite variable [32]. Searching for compounds that can induce alternative cell death pathways may better contribute to target different cellular functions and exploit synergistic effects not yet discovered. 

## 3. Potential Medical Applications for Yessotoxin

**Figure 1:**
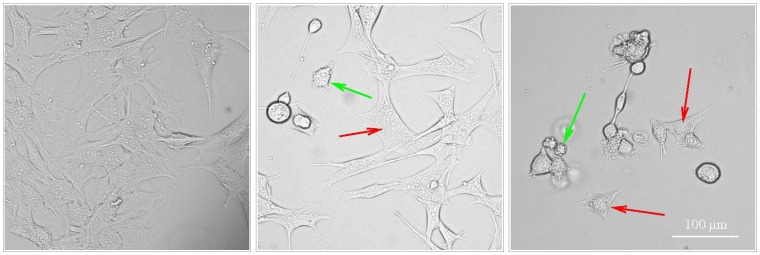
Activation of apoptosis and paraptosis like-cell death in BC3H1 myoblast cell lines to 100 nM YTX. Left: untreated cells. Centre and right: exposed cells after 48 h and 72 h (respectively) showing morphological traits of apoptosis (green arrow) and paraptosis (red arrow). Scale bar: 100 µm.

**Figure 2:**
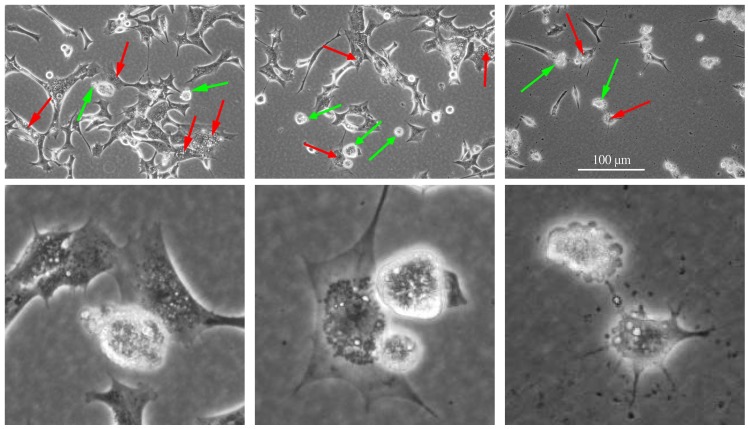
Apoptotic and paraptotic features in BC3H1 myoblast cell lines exposed to 100 nM YTX. Exposure times are 24 h (left), 48 h (centre) and 72 h (right). Membrane blebbing and apoptotic bodies (green arrow), cytoplasmic vacuolation (red arrow). Note that apoptotic and paraptotic features are already evident at 24 h YTX exposure. Each of the lower image is an enlarged subset of the image above. Scale bar: 100 µm.

YTX can activate different cell death programmes in BC3H1 myoblast cell lines [19,23]. Figure 1, Figure 2 illustrate apoptotic and paraptotic traits in BC3H1 cells due to YTX exposure. Membrane blebbing and formation of apoptotic bodies are typical for apoptosis while extensive cytoplasmic vacuolation is a trait for paraptosis. This variability indicates potentials to compose synergistic effects of interest within development of therapeutic methods to avoid drug-resistance in cancer cells. 

Korsnes *et al*. [19] and Korsnes *et al.* [23] provide light and electron microscopy pictures showing morphological traits typical to apoptosis and paraptosis in BC3H1 cells subject to YTX exposure during 24, 48 and 72 h. Typical morphological traits of apoptosis in this case were membrane blebbing, nuclear skrinkage and formation of apoptotic bodies. Caspase 9 activation was detected by western blotting and immunofluorescence labelling. DNA agarose gel electrophoresis and TUNEL assay evidence lack of DNA fragmentation. One similarly found excessive cytoplasmic vacuolation, no DNA fragmentation, no PARP cleavage, uncondensed chromatin, mitochondria and ER swelling and JNK activation as typical paraptotic traits. Double membrane vacuoles or autophagosomes in the cytoplasm of dying cells may be present during YTX exposures. However, formation of autophagosomes is not sufficient to incriminate autophagy as a mechanism of cell death [39]. Autophagosomes may accumulate not due to activation of the flux through the autophagic pathway; rather, they may appear as a result of inhibition of their maturation process [39]. Autophagosomes may also be involved in elimination of damaged mitochondria, damaged portions of the ER membrane, or selective removal of functionally redundant organelles [40,41]. Paraptosis and autophagic cell death can not be easily distinguished with standardised *in vitro* biochemical assays [42]. Induction of autophagic cell death under YTX insult has yet to be demonstrated. 

Sperandio *et al.* [24] reported that cell death insults can produce a two-pronged response leading either to activation of the apoptotic pathway or to activation of the paraptotic pathway. Activation of a specific cell death pathway can depend on a variety of factors including cell type, the hierarchy of inter-cellular organelles and the type and severity of insult [2,24]. Multiple pathways might interact to execute cell death, although the resulting morphological appearance would be decided by the metabolic situation, the activation or suppression of individual subroutines or the relative speed of their execution in each cell type [43,44,45,46,47]. Moreover, a specific cell death-related phenomenon may occur along with the execution of another cell death mode or a cell death-associated biochemical process can occur at a sub-lethal or transient level, which does not lead to the cell demise [4]. Crosstalk between different cell death subroutines can occur under stress conditions and it may result in the activation of multiple lethal mechanisms, which can exhibit different degree of overlap. Interference between pro-survival and pro-death pathways may determine if and by which subroutine the cell will eventually die [4]. 

Receptors involved in mediating cell death may also execute the apoptotic or the paraptotic pathway, or both pathways [24]. Furthermore, the contribution of individual receptors could also be relevant in the decision of the mode of cell death [44]. Activation of the apoptotic and the paraptotic pathway in BC3H1 myoblast cell lines under YTX insult might be related to individual receptors in such cell lines and the interaction of YTX with different cellular components that are involved in the expression and function of such cellular receptors. 

YTX is already suggested for different therapeutic approaches [48,49]. López *et al.* [48] proposed to use YTX as an anti-tumour drug, and López *et al.* [49] proposed to use YTX and its analogues to treat and/or prevent neurodegenerative diseases such as the Alzheimer’s disease. The Alzheimer’s disease is characterized by abnormal accumulation of β-amyloid and TAU proteins. Misfolded β-amyloid and TAU proteins form aggregates and adversely affect normal cellular function in the neurons where they induce a progressive degeneration. López *et al.* [49] brought the idea that YTX may help to avoid this pathological accumulation of proteins. They showed that YTX at a low concentration of 1 nM can decrease levels of the TAU and the β-amyloid protein in transgenic mice. These different and opposite therapeutic approaches indicate that YTX concentration and cell-specificity might be critical for the induction of different cell death mechanisms. Therefore, identification of chemical compounds that can modulate induction of apoptosis and inhibition of paraptosis would be beneficial for possible therapeutic applications. 

Evaluation of programmed cell death induction by YTX with different model cellular systems will increase understanding about YTX’s mechanisms of action and cross-talk among signalling pathways involved in cell death. For example, induction of the apoptotic cell death receptor pathway under YTX exposure is still undetermined and it could be a new potential therapeutic use regarding the apoptotic pathway. Targeting apoptosis by employing the death receptor pathway constitutes a potential therapeutic strategy in many cancer cells, since cancer cells are known to their resistance to apoptosis induction. 

## 4. Other Examples of Complex Induction of Programmed Cell Death

There are currently few reports of chemical agents which can activate both apoptosis and paraptosis in cells. Table 1 summarises such observations. The table includes a list of different insults which can activate different cell death programmes. The induction seems to be cell-specific. Multiple pathways may interact to form a complex system of positive feedback loops to execute cell death. The resulting morphology would be decided by the metabolic situation, intracellular protein localisation, transport inside the cells, the activation or suppression of individual subroutines or the relative speed of their execution in each cell [43,45,46,47,50]. Induction of multiple pathways may be necessary to ensure the removal of injured cells or prevent survival of unhealthy cells. It seems likely that accumulation of damage incompatible with cell survival would require disruption of several vital functions. Therefore, the progression of the death programmes and the safe disposal of the injured cell may appear as a positive feedback mechanism to remove such unwanted cells [50]. 

**Table 1:** Examples of multiple induction of cell death programmes under different insults.

Reference	Cell type	Insult	Pathway	Mediated by
Sperandio *et al.* [24]	293T cells, Apaf-1 null mouse embryonic fibroblast	Transfection with the human insulin-like growth factor I receptor (IGFIR)	apoptosis, paraptosis	caspase-9, Apaf-1 independent
Kanasaki *et al.* [51]	Rat Pituitary GH3 Cells	Bromocriptine	apoptosis	p38
Palmeri *et al.* [52]	Experimental rat pituitary tumours		paraptosis	p38, ERK1/2, PKCδ
Wang *et al.* [53]	HEK293, HeLa and 293T cells	Transfection with the TAJ/TROY (novel member of the TNFR family)	paraptosis-like	overexpression of programmed cell death 5 (PDCD5)
Samadder *et al.* [54]	MEFs (mouse embryonic fibroblasts)	Glycosylated anti-tumour ether lipids (GAELs)	paraptosis-like	mTOR-independent (mammalian target of rapamycin)
Li *et al.* [55]	HCT116 (colorectal cancer cells)	Ginsenoside RH2	apoptosis, paraptosis-like	caspase-3 activation and p53
Korsnes *et al.* [19]	BC3H1 myoblast cell lines	Yessotoxin	apoptosis	caspase-3 activation
Korsnes *et al.* [23]			paraptosis-like	caspase-9 activation, JNK/SAPK1
Asare *et al.* [56]	Hepa1c1c7 cells	1-Nitropyrene (1-NP)	apoptosis, paraptosis	ERK1/2, p38, JNK
Yoon *et al.* [57]	MDA-MB-231, MDA-MB-435S and Hs578T (breast cancer cells)	Curcumin	paraptosis-like	ERK, JNK
Zhang *et al.* [58]	human colon carcinoma SW620 cells	δ-tocotrienol	paraptosis-like	Suppression of the Wnt signalling pathway.

Simultaneous activation may occur under the same insult and in the same cell population [24,55,56]. Insults here may produce synergistic effects mediated by regulators of several pathways. However, the relationships between the pathways remain to be clarified. The induction apparently depends on cell type, type of insult, exposure time, type of cellular receptors and possibly unknown factors. 

It has been suggested that multiple cell death programmes can be activated in the same cell population. The dominant cell death phenotype is determined by the relative speed of the available cell death programmes. Although characteristics of several cell death pathways can be displayed, only the fastest and most effective death pathway is usually evident [1,59]. However, Chi *et al.* [60] suggested that a cell may switch back and forth between different death signalling pathways. This may produce apparent display of characteristics from several pathways. The evolutionary advantage of activation of multiple cell death pathways may be a more efficient and selective elimination of damaged cells to protect the organism against the development of malignant diseases. This fact may have general medical interest for the development of new therapeutic applications. 

Sperandio *et al.* [24] reported that out of the 7075 differentially expressed genes evaluated in a micro-array analysis, less than 2 percent are common to apoptotic and paraptotic cell death programmes. These programmes probably overlap and share signalling pathways. It may therefore be difficult to classify and to distinguish between them. 

Screening for novel molecules that can trigger simultaneously programmed cell death programmes may be a current pharmacological challenge. However, the absence of model systems that can be useful for the identification of the molecular mechanisms involved in different types of programmed cell death has limited their identification. 

## 5. Concluding Remarks

Many natural chemical compounds can at low concentrations interfere with well-conserved cell signalling pathways. This ability makes them a resource for future therapeutics. Yessotoxin is such a natural small molecule compound which can induce distinct programmed cell death mechanisms in several model cell systems. The induction seems to be concentration-dependent and cell-specific. The actual cell death modalities may involve cross-talk between several signalling pathways. This is though still not clarified [22]. Knowledge about how different cell systems respond to YTX may clarify underlying mechanisms of toxicity and help to identify novel molecular mechanisms that could be tested for therapeutic applications of medical relevance. 
